# Effect of a governmentally-led physical activity program on motor skills in young children attending child care centers: a cluster randomized controlled trial

**DOI:** 10.1186/1479-5868-10-90

**Published:** 2013-07-08

**Authors:** Antoine Bonvin, Jérôme Barral, Tanja H Kakebeeke, Susi Kriemler, Anouk Longchamp, Christian Schindler, Pedro Marques-Vidal, Jardena J Puder

**Affiliations:** 1 Institute of Sport Sciences, University of Lausanne, Quartier Unil - Mouline, Bâtiment Geopolis, Lausanne 1015, Switzerland; 2 Child Development Center, University Children’s Hospital, Zürich 8032, Switzerland; 3 Swiss Tropical and Public Health Institute, University of Basel, Socinstrasse 57, Basel 4002, Switzerland; 4 Health League, Rue de la Mouline 8, Chavannes-près-Renens 1022, Switzerland; 5 Institute of Social and Preventive Medicine, University of Basel, Basel, Switzerland; 6 Service of Endocrinology, Diabetes and Metabolism, Centre Hospitalier Universitaire Vaudois, University of Lausanne, Rue du Bugnon 46, Lausanne 1011, Switzerland

**Keywords:** Physical activity, Motor skills, Child care centers, Randomized controlled trial, Government

## Abstract

**Objective:**

To assess the effect of a governmentally-led center based child care physical activity program (Youp’là Bouge) on child motor skills.

**Patients and methods:**

We conducted a single blinded cluster randomized controlled trial in 58 Swiss child care centers. Centers were randomly selected and 1:1 assigned to a control or intervention group. The intervention lasted from September 2009 to June 2010 and included training of the educators, adaptation of the child care built environment, parental involvement and daily physical activity. Motor skill was the primary outcome and body mass index (BMI), physical activity and quality of life secondary outcomes. The intervention implementation was also assessed.

**Results:**

At baseline, 648 children present on the motor test day were included (age 3.3 ± 0.6, BMI 16.3 ± 1.3 kg/m^2^, 13.2% overweight, 49% girls) and 313 received the intervention. Relative to children in the control group (n = 201), children in the intervention group (n = 187) showed no significant increase in motor skills (delta of mean change (95% confidence interval: -0.2 (−0.8 to 0.3), p = 0.43) or in any of the secondary outcomes. Not all child care centers implemented all the intervention components. Within the intervention group, several predictors were positively associated with trial outcomes: 1) free-access to a movement space and parental information session for motor skills 2) highly motivated and trained educators for BMI 3) free-access to a movement space and purchase of mobile equipment for physical activity (all p < 0.05).

**Conclusion:**

This “real-life” physical activity program in child care centers confirms the complexity of implementing an intervention outside a study setting and identified potentially relevant predictors that could improve future programs.

**Trial registration:**

Clinical trials.gov NCT00967460

## Background

Participating in an adequate amount of physical activity is beneficial for the health of children (e.g. social development, obesity prevention, bone health) [1-3]. Over the past two decades, physical activity among children and adolescents has been decreasing at an alarming rate [4]. The causes of this complex problem are multifactorial. Less developed motor skills have been identified as a potential contributor to physical inactivity in young children [3]. Relationships between physical activity and motor skills are bidirectional and both physical activity and motor skills represent essential components for developmental processes of children and influence motor, cognitive or emotional aspects of children’s health [3,5]. Thus, increasing physical activity provides more opportunities to promote neuromotor development [6,7], which reinforces motor competences. On the other hand, well-developed motor skills contribute to children’s propensity to engage in physical activity [5,8]. Stodden et al. [3] suggest that the impact of physical activity or of motor skill competences is especially pronounced in young children. The more they are involved in active behaviors, the more they built a sufficiently adapted motor repertoire for specific movement contexts. In contrast, limited physical activity can contribute to impaired motor skills and motor coordination in older children which may in turn lead to poorer self-efficacy and lower life satisfaction [5,9]. Physical activity interventions in children have shown to have positive impact on self-esteem [10] and the effect of physical activity on quality of life is especially pronounced in obese children [1,11]. As the first years of life are crucial in determining later lifestyle behaviors and health [12-15], child care centers have been identified as important settings to deliver physical activity interventions to improve motor skills and prevent obesity [16-20]. They offer the advantage of involving a large number of young children and their educators are receptive for training [21]. Further, providing a supportive social environment and rearranging the existing built environment of the child care center can represent an attractive feasible low-cost non-curricular approach to increase physical activity [20]. However, a recent Cochrane collaboration review [22] has identified a need to study physical activity intervention in preschoolers. To our knowledge, few randomized preschool-based physical activity interventions have been performed [2,23-35]. There exist even fewer child care-based interventions targeting younger children, e.g. children aged 2–4 years [36,37]. Of the existing four controlled child care-based studies, two included slightly older children [30,34] (mean age 4.2 and 4.1 years respectively), one was small-sized and of short duration [37] (n = 42, 8 weeks duration), one was not randomized and did not measure physical activity [36] and two did not evaluate motor skills.

To fill the existing gap, our objective was to conduct a cluster randomized controlled trial (RCT) taking advantage of the implementation of a “real-life” physical activity program in child care centers (“Youp’là Bouge”) carried out by the local governmental institutions. We thus hypothesized that the program would improve motor skills using age-specific motor tasks [38,39]. In addition, the study focused on possible effects on children’s body mass index (BMI), their child-care-based physical activity level and their quality of life. Based on results of previously published studies [40-44], our aim was also to evaluate the impact of certain predictors that have been associated with improved outcomes within a physical activity intervention.

## Methods

### Study design, setting and participants

This cluster-RCT was performed in 3 out of 6 cantons in the French-speaking part of Switzerland (Vaud, Neuchâtel and Jura). A canton represents a geographic government area. Vaud has 725’000, Neuchâtel 174’000 and Jura 70’000 inhabitants with all three cantons having both rural and urban areas. The cantonal ethical committees approved the study and parents gave informed written consent. The RCT was registered as clinical trials.gov NCT00967460.

**Figure 1:**
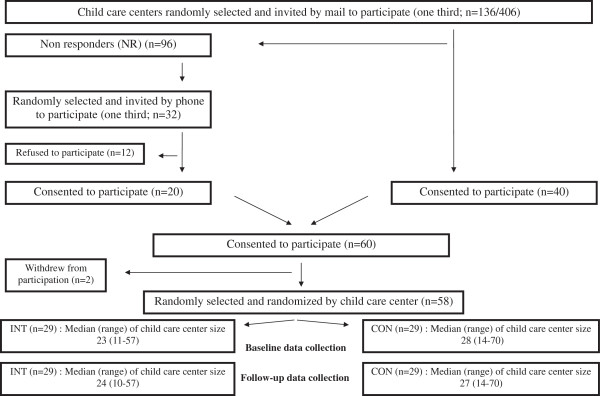
**Trial profile of clusters.** INT = Intervention child care centers, CON = Child care centers. All public child care centers in the three cantons were eligible to participate in the program. No precise information was given concerning the nature of the two withdrawals.

As the respective cantonal governmental institutions conducted the intervention, all public child care centers in these cantons were eligible. Recruitment, selection and a blinded randomization of the child care centers took place between October 2008 and February 2009 and were performed by a governmental coordinator not involved in the assessment of the program. For organizational purposes, the governmental agencies decided to select and randomize all the centers before the end of the school year in order to be able to start the intervention after the summer break. A third of the public child care centers (n = 136/406) in the three cantons were randomly selected and invited by mail to participate in the Youp’là Bouge physical activity program (Figure 1). Two child care centers withdrew at the beginning of the program without giving more information. Fifty-eight child care centers from rural and urban areas participated in the program. They were 1:1 assigned to a control (n = 29, corresponding to a waiting list for a future participation) and an intervention (n = 29) group. Educators, parents and children were informed that the intervention aimed to promote children’s health, but were unaware of the main objectives of the study.

### Intervention

The physical activity intervention lasted from September 2009 to June 2010 and was designed to intervene at the individual (children, educators, and parents) and environmental (child care built environment; physical activity as part of the daily program) level, based on a socio-ecological conceptual model [45]. Behavioral strategies, targeting children, educators and families aimed to improve knowledge about physical activity benefits and to increase pleasure, self-efficacy and skills and to integrate physical activity into the daily life of the child care [46]. The physical activity intervention also integrated several components that have been previously shown to be effective [22] and that can be implemented on a large scale: 1) Training and support of the educators [42,47]; 2) Rearrangement of the child care built environment [48]; 3) Encouragement of parental involvement [49]; 4) Recommendation of daily physical activity [48]. However, in order to attract as many child care centers as possible and respect their specific needs for autonomy, no precise mandatory demands were made regarding the daily physical activity time or the use of a structured physical activity curriculum.

### Training and support of the educators

Five workshops providing theoretical and practical physical activity support were held for the educators of the respective intervention child care centers between April 2009 and July 2009. At least one educator per child care had to be present, but child care centers were encouraged to train more than one educator. Workshops were given by the coordinator, by sport scientists specialized in physical activity and health and by physicians. Themes of the workshops were 1) “Movement and motor development”: Educators were made aware about the importance of physical activity and of motor development for global childhood development. 2) “Moving - a pleasure and a need”: Educators learned to better understand the main factors related to inactivity and how to promote physical activity in young children and families. Practical aspects were also given, especially about the use of the materials. 3) “Practical aspect of physical activity”: The trainers highlighted the importance of letting the children move freely around and autonomously explore the environment. The aspect of security was also treated in this workshop. 4) “Health promotion in child care centers”: Educators learned more about the health implications of physical activity/inactivity (such as obesity) and the importance of involving the parents in such a program. 5) “Implementation of the project”: Educators were given tools showing how to implement the program in their specific context. The coordinator provided the child care centers with flyers, documentation and personal support for the parental sessions.

During the intervention, the coordinator organized every 2 months regular group meetings in the different child cares with the trained educators of all child care centers of a canton. During the meetings, the child care presented what they had changed, exchanged ideas and discussed their barriers, problems and achievements. The coordinator was available for any questions and concerns that occurred between the meetings.

### Child care environment

Each child care center received a budget of $1500 for the rearrangement of their environment. The coordinator advised educators on how to make the child care environment more activity-friendly by specifically recommending providing an indoor movement space and provision of portable or/and fixed indoor or outdoor physical activity equipment.

### Parental involvement

Child care centers were encouraged to involve parents in an information and discussion session presenting the program and the benefits of physical activity and to exchange views on how to integrate physical activity in their family environment. In addition, the parents of all intervention child care centers received flyers containing information regarding Youp’là Bouge.

### Control group

The control group did not receive any intervention and continued their regular program. No financial incentives were provided for the participants of either group.

### Data collection procedures and measures

We tested the efficacy of the program by comparing participants allocated to the intervention group with those in the control group at baseline and after 9 months. Trained researchers blinded to group allocation provided the assessments.

### Primary outcomes

Motor skills were chosen as the primary outcome of this low-level intervention based on the results of a previous intervention in a similar setting [34]. Motor skill measures were adapted from the Zurich Neuromotor Assessment (ZNA) test, a standardized and reliable test for 5- to 8-year-old children [50,51]. Based on the developmental stages (initial, elementary and mature) according to Gallahue et al. [52], this test has been recently extended and subsequently validated for 3- to 5-year-old children [38,39,53]. Two of the three evaluators of the present study conducted a pilot study in two additional child care centers in order to compute the test-retest reliability (n = 33, Intra class correlation: r = 0.5, p < 0.05 for both evaluators over a two weeks period) and the inter-rater correlation (n = 42, Intra class correlation: r = 0.8, p < 0.05) of the “global motor score” (see below).

**Figure 2:**
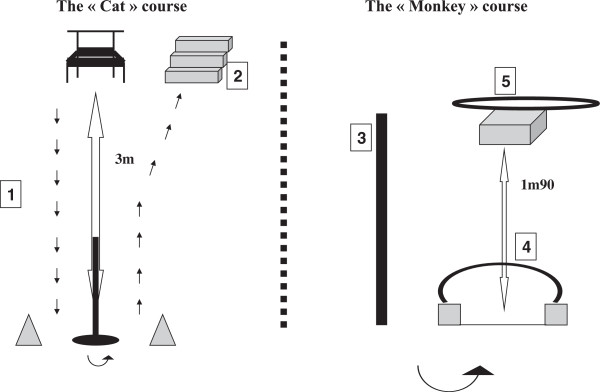
**Description of the obstacle courses. 1)** Running **2)** Climbing up and down the stairs **3)** Balancing **4)** Getting up **5)** Landing after jumping.

In this test, five motor skill tasks (climbing up and down the stairs; running; balancing; getting up; landing after jumping; no 1–5 in Figure 2) were tested using two playful obstacle courses (the “Cat” and the “Monkey”). Each task was rated on a 5-point scale scoring from 0 for worst to 4 for best. Motor skills’ testing was carried out in a separate room in groups of 4 to 6 children in the presence of 1 educator and 3 evaluators. Each of both obstacle courses was explained and demonstrated to a subgroup of 2 to 3 children and each child was evaluated and scored individually by one evaluator. This test allows assessing motor skills of 6 children with two evaluators within 15 minutes. A “global motor score” (ranging from 0–20) was calculated summing up the score of each motor skill task.

Children that refused to participate in even one of the five motor tasks were removed from all analyses to be able to use a “global motor score” (n = 115; 18% refusals). Refusals were especially numerous in younger children (mean age of refusals: 2.8 ± 0.6 yrs).

### Secondary outcomes

Standing height was determined and body weight was measured using an electronic scale (Seca, Basel, Switzerland; accuracy 0.05 g). Children were classified into two BMI-groups (normal weight and overweight group (including both overweight and obese children)) according to the International Obesity Task Force criteria [54].

We also aimed to determine if the intervention demonstrated an effect on children physical activity levels during child care. Due to cost and limited human resources, 30 of the 58 child care centers were randomly selected after stratification for group assignment to also include physical activity measurements which were carried out one week after the other outcomes. Physical activity was measured over one day at the child care center with an accelerometer (GT1M, Actigraph, Florida, USA). The accelerometer was worn around the hip and programmed to save data in 15 s intervals (epoch size of 15 s), as proposed and validated for this age [55-57]. Data were considered valid if collected for at least 3 hours. This allowed the inclusion of children attending the child care center during half days. Mean total wearing time was 6.1 hours (standard deviation (SD) 1.4). Sequences of at least 10 min of consecutive zero values were removed and interpreted as accelerometer not worn [58]. Average physical activity level was expressed in counts per minute (cpm, total counts recorded divided by total daily wearing time). Physical activity was further categorized using age-specific cut-offs [57] into moderate-vigorous physical activity (MVPA; ≥420 counts/epoch) and vigorous physical activity (VPA; ≥842 counts/epoch). Thereby, data are expressed as the number of epochs/hour above the respective cut-offs.

Quality of life of the participating children was assessed using the parent report for children of PedsQL 4.0 Generic Core Scales questionnaire validated for this age group [59]. Information about parental socio-cultural cofounders (migrant status, education and workload) were obtained through a general health questionnaire [33,60-62] which was filled out at home. When necessary, the educators gave help to the parents to fill it out. Country of birth determined parental migrant status. Being migrant was defined as born outside of Switzerland and educational level as the highest school grade completed (5 levels) [61,63].

### Process evaluation and predictors

Process evaluation of the implementation in all 29 intervention child care centers was performed by the program coordinator for the following 6 predictors: 1)* Number of trained educators per child care center* (child care centers included between one to eight educators for this age group); 2) *educators’ motivation*; 3) *management’s involvement* (the latter two on a 3-point Likert scale: 1-Hardly motivated/involved 2-Moderately motivated/involved 3-Strongly motivated/involved); 4) *child care environment*: free access to a movement space (yes/no); 5) *type of equipment bought* (mobile only versus mobile and fixed for indoors and/or outdoors, respectively); 6) *parental involvement*: organization of a information and discussion session with parents (yes/no). These predictors were determined based on previous cross-sectional studies that notified their association with physical activity [40-44]. The coordinator also intended to evaluate the changes of time dedicated to daily physical activity, but educators were unable to provide exact information. The process evaluation was used to assess the impact of these parameters (predictors) on the chosen outcome changes.

### Satisfaction

At the end of the intervention, parents and educators in the intervention group were asked to fill out a questionnaire determining their satisfaction with the program. The degree of satisfaction of the program was obtained from a 4 points Likert scale question: 1- highly satisfied 2- satisfied 3- more or less satisfied 4- unsatisfied. Parents also had the possibility to provide any critical comments about the program.

### Statistical methods

All analyses were performed using Stata version 11.0 (Statacorp, College Station, TX, USA). For the power calculations we assumed an intraclass correlation of 0.10, i.e., corresponding to a random class effect whose standard deviation is 0.34 times the standard deviation of the primary outcome measure (motor skills) within classes. We also assumed that on average 10 children per child care center would be available for baseline and follow-up measurements (due to non-participation, attrition, moving, sickness or planned absence on the testing day) and that the intervention effect would be ≥0.4 standard deviations for the main primary outcome. Under these conditions, we calculated that 28 child care centers per group would provide 90% power with a p-value < 0.05, if the true treatment effect equals at least 0.4 sigma. In total, 29 child care centers per group were included in order to account for possible attrition. Analyses were performed on an intention to treat basis; using individual children data but adjusting for clustering of outcomes within child care centers. Results are described as mean standard deviation (SD) or percentages, as data were normally distributed. Baseline characteristics between the intervention and control group were compared by mixed linear or regression models. Intervention effects were estimated using mixed linear regression models with the change in the respective outcome as the dependent variable, adjusting for baseline outcomes, age and sex as covariates. In case of binary outcome variables, logistic regression models were used with the follow-up outcome as the dependent variable adjusting for the same covariates including the respective baseline values. The effect estimates for quantitative outcomes are expressed as the difference between the mean individual changes in the intervention and the mean individual changes in the control groups. The effect estimates for binary outcomes were obtained from logistic regression models and are presented as odds ratios with 95% confidence intervals. All analyses were also adjusted for migrant status and educational level. No p-value adjustment for parallel comparisons was made for the secondary outcomes because the focus was on effect estimation and there is considerable correlation between the outcome and the predictor variables considered. In order to be able to further improve the program in the future, we performed exploratory analyses to test if the potential predictors mentioned in the process evaluation would be associated with improved longitudinal outcomes within the intervention group. Mixed linear models were used with motor skills, BMI, physical activity and quality of life as the respective dependent outcome variables and either one or all 6 predictors (see “process evaluation and predictors”) as the respective predictor variables adjusting for the baseline outcomes, age and sex as covariates. We thereby compared those child care centers that implemented these items to those who did not. For all analyses, there was no imputation of missing data.

## Results

### Study sample

**Figure 3:**
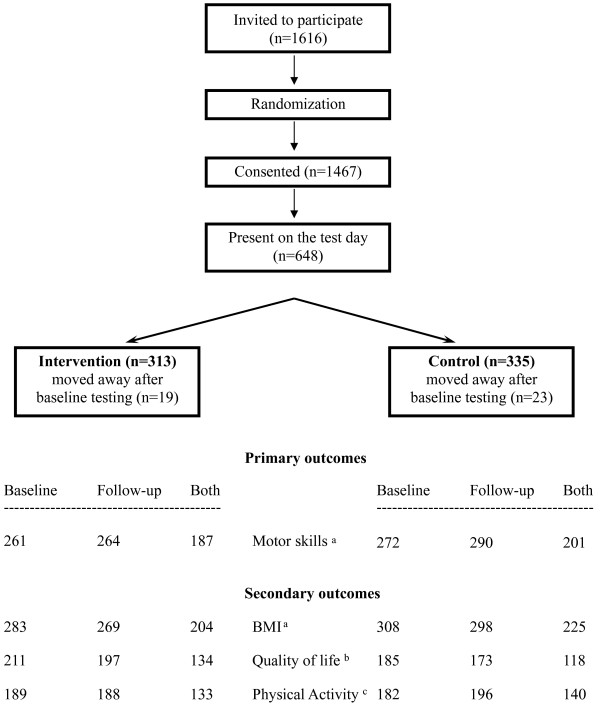
**Trial profile of participants. **^1^Due to a mean attendance of the children at child care of 48%, 648 children were present on the test day at baseline, 589 with valid BMI and 533 with valid motor skill (global motor score) measures. ^2^Valid data for total quality of life as assessed by PedsQL questionnaire. ^3^Due to cost and logistic reasons, 30 of the 58 child care centers were randomly selected to also include physical activity measurements which were performed one week after the other outcomes.

Participant flow is shown in Figures 1 and 3. A total of 58 child care centers (n = 1616 children) entered the program. Informed consent was obtained from 91% of the parents’ children (n = 1467). Of those, a total of 737 children received the intervention. Due to a mean attendance of the children at child care center of 48%, the current sample focuses on the 648 children who were present on the test day (313 intervention and 335 controls). None of the child care centers left the program, but 19 children (6%) in the intervention group and 23 (7%) in the control group had moved away by the end of the year. Child and parental baseline characteristics according to study group are shown in Table 1. Mean age of the children was 3.3 years (SD 0.6). No significant differences were observed in baseline characteristics and outcome variables between the control and the intervention groups (Table 1, all p > 0.09).

**Table 1:** Child and parental baseline characteristics according to study group

**Characteristic**	**Control**	**Intervention**	**Total sample**
n	335	313	648
Gender, male, % (n)	49 (164)	51 (171)	51 (335)
Age, mean ± SD, y (n)	3.3 ± 0.6 (335)	3.4 ± 0.6 (313)	3.3 ± 0.6 (648)
Weight, mean ± SD, kg (n)	15.6 ± 2.2 (314)	15.8 ± 2.3 (283)	15.7 ± 2.2 (597)
Height, mean ± SD, cm (n)	97.9 ± 6.5 (308)	98.1 ± 6.4 (283)	98.0 ± 6.4 (591)
BMI, mean ± SD, kg/m^2^ (n)	16.2 ± 1.2 (308)	16.3 ± 1.4 (283)	16.3 ± 1.3 (591)
Overweight children, % (n) ^a^	11 (34)	15.7 (44)	13.2 (78)
Parental migrant status, % (n) ^b^	61.3 (146)	54.7 (145)	57.9 (291)
Parental low educational level, % (n) ^c^	15.8 (35)	19 (49)	17.5 (84)

^a^ according to the International Obesity Task Force criteria.

^b^ migrant status was defined as at least one parent born outside of Switzerland.

^c^ parental low educational level was defined as at least one parent with 9 years or less of education.

No significant differences were observed in baseline characteristics and outcome variables between the control and the intervention groups (all p > 0.09).

### Outcomes

The results of the intervention on primary and secondary outcomes are summarized in Table 2. There was no intervention effect on the primary (motor skills) or the three secondary outcomes (BMI or the prevalence of overweight, measured physical activity and quality of life). Adjusting for parental migrant status or educational level did not alter the results (not shown).

**Table 2:** Baseline and follow-up values of primary and secondary outcomes and intervention effects

***Outcomes***	**Baseline**		**Follow-up**		**Effect estimates**	
	***Intervention***	***Control***	***Intervention***	***Control***		
	***n = 313***	***n = 335***	***n = 280***	***n = 308***		
*Primary outcome*					*Δ of mean change (95% CI)*^a^	*p value*
Motor skills (Global motor score), mean ± SD	12.4 ± 3.5	12.5 ± 3.5	14.2 ± 2.9	14.2 ± 2.8	−0.2 (−0.8 to 0.3)	*0.43*
*Secondary outcomes*						
Average PA, mean ± SD, counts/min	620 ± 278	600 ±206	765 ± 340	711 ± 219	55.9 (−30.6 to 142.3)	*0.21*
MVPA, mean ± SD, epochs/hour ≥ 420 counts	29.2 ± 14	28.1 ± 12.5	37.2 ± 17.1	35.9 ± 13.7	1.1 (−4.2 to 6.4)	*0.68*
VPA mean ± SD, epochs/hour ≥ 842 counts	8.1 ± 6.1	7.4 ± 5.3	10.3 ± 7.5	9.2 ± 6.2	1.22 (−1.25 to 3.69)	*0.33*
Quality of life (PedsQL™ Score), mean ± SD	83.0 ± 9.1	82.0 ± 11.0	82.9 ± 9.3	81.5 ± 11.3	1.1 (−1.8 to 3.9)	*0.46*
BMI, mean ± SD, kg/m^2^	16.3 ± 1.4	16.2 ± 1.2	16.1 ± 1.3	16.2 ± 1.3	−0.7 (−0.2 to 0.6)	*0.29*
					*Odds Ratio (95% CI)*^b^	
Normal Weight, %	84.3	89.0	82.5	85.9	0.74 (0.48 to 2.76)	* 0.2*
Overweight, %	15.7	11	17.5	14.1		

*BMI* body mass index*, PA* physical activity*, MVPA* moderate to vigorous physical activity*, VPA* vigorous physical activity*, CI* confidence intervals*.*

^a^In the case of quantitative outcome variables, the effect estimates describe the difference between the mean individual changes in the intervention group and the mean individual changes in the control group using mixed linear models and adjusting for the respective baseline values, age, gender and for the cluster factor child care center (i.e. the unit of randomization).

^b^In the case of the binary outcome variables, effect estimates were obtained from mixed logistic regression models with the same adjustments, and they are expressed as odds ratios.

n is based on the number of children present at the test day. See Figure 3 for more details on the number of valid outcome data at a given time point.

### Process evaluation, predictors and satisfaction

All intervention centers provided at least one, and five centers (17%) two or more educators for training. These educators attended all workshops. The educators were either strongly (50%) or moderately (50%) motivated. The management was either strongly (70%) or moderately (30%) involved. All intervention centers rearranged their indoor environment and purchased physical activity indoor equipment (69% of it mobile), while 28% also purchased outdoor equipment (only mobile); 69% of the centers provided free access to a movement space and 72% organized an information session with parents (i.e. parental involvement). Table 3 shows the predictors with significant impact on the assessed outcomes within the intervention group: Child motor skills were higher where centers offered a parental information session or free access to a movement space, while the motivation and training of additional educators was associated with higher effects on BMI. The purchase of only mobile as compared to mobile and fixed indoor equipment and providing a free access to a movement space was associated with higher average physical activity and more intense physical activities (VPA and MVPA, respectively). No impact on the chosen outcomes was found for the other potential predictors (all p > 0.05, data not shown). When including all six predictors in addition to sex, age and the respective outcome variables in multivariate analyses, the above mentioned results (Table 3) remained unchanged except for two predictors: The association of a parental information session with increased motor skills (p = 0.1) and the presence of mobile indoor equipment (as compared to mobile and fixed indoor equipment) with greater average physical activity (p = 0.08) did not remain significant. However, mobile equipment continued to be significantly associated with VPA and newly also on MVPA, when all predictors were taken simultaneously into account.

**Table 3:** Significant predictors of the primary and the secondary outcomes

**Predictors **^**a**^	**% of child care centers **^**b**^	**Outcomes**	**Effect estimates of the predictors**	
			***Δ of mean change (95% CI)***^***c***^	***p value***
Information session with parents	72%	Motor skills (Global motor score)	1.15 (0.16 to 2.13)	*p = 0.02*
Free access to a movement space	48%	Motor skills (Global motor score)	0.92 (0.12 to 1.70)	*p = 0.02*
High motivation of the educators	59%	BMI (kg/m^2^)	−0.16 (−0.30 to −0.01)	*p = 0.04*
Training of additional educators	17%	BMI (kg/m^2^)	−0.26 (−0.50 to −0.01)	*p = 0.04*
Free access to a movement space	48%	Average PA (counts/min)	144 (41 to 248)	*p = 0.006*
Free access to a movement space	48%	MVPA (epochs/hours ≥ 420 counts)	9.4 (2.9 to 15.9)	*p = 0.005*
Purchase of mobile indoor equipment	69%	Average PA (counts/min)	170 (12 to 328)	*p = 0.03*
Purchase of mobile indoor equipment	69%	VPA (epochs/hours ≥ 842 counts)	4.0 (0.1 to 7.9)	*p = 0.04*

*BMI* Body Mass Index, *PA* physical activity, *VPA* vigorous physical activity, *CI* confidence intervals.

^a^ Feedback was obtained by the program coordinator from all 29 intervention child care centers at the end of the intervention. Only the significant predictors are shown in this table (5 out of 6).

^b^ Percentage of child care centers that provided the respective predictors.

^c^ The effect estimates describe the difference between the mean individual changes in the intervention child care centers fulfilling the respective criterion and the mean individual changes of the remaining intervention child care centers using mixed linear models and adjusting for the respective baseline values, age, gender and for the cluster factor child care center (i.e. the unit of randomization).

All educators of the intervention child care centers were either highly satisfied (43%) or satisfied (57%). All parents were highly satisfied with the different parts of the program, but 10% would have wished a faster and more extensive return of the results and 3% a higher parental involvement.

## Discussion

The Youp’là Bouge program, a governmentally-led “real-life” physical activity program in child care centers, did not lead to improvements in child motor skills. The intervention also resulted in smaller effect sizes for BMI, child care-based physical activity and quality of life than were hypothesized. This program was intended to represent a low-level feasible physical activity intervention integrating several components that have been shown to be effective in previous lifestyle interventions [22] namely a daily physical activity period in the child care center, training and support of the educators, rearrangement of the child care built environment and encouragement of parental involvement. The design aimed to identify potentially relevant and modifiable predictors of improved outcomes. We thereby provide results of a program that was studied as a RCT, yet not conducted by a study group, but initiated and led by the local governmental education and health institutions.

### Comparison with other studies

Previous published preschool-based randomized controlled trials had studied the impact of physical activity interventions on motor skills in typically developing healthy preschoolers and all of them were investigator-driven studies (as opposed to governmentally-led “real-life studies”) [24,29,32,35]. Mean age of the children was well over 4 years (ranging from 4.4 to 6.1 years) compared to 3.3 years in our program. All offered a relatively intense program that included structured physical activity ranging from twice to 4 times a week and lasting 6–10 months. All except for one study lead to an improvement of motor skills, as measured by fundamental motor skills testing (jumping, balance, skipping, and ball exercises) or agility. The study [24] that did not improve motor skills offered 30 min/day of vigorous physical activity games and measured side-to-side jumps from the Karlsruher Motor Screening 3–6 [64] as their measure of motor skills. To our knowledge, only 2 randomized controlled trials were child care-based and evaluated motor skills, both of them being investigator-driven [30,34]. The mean age of the children was over 4 years and thus higher than the mean age in our program. Both studies included structured sessions of physical activity three to four times/week ranging from 30–45 minutes over around 6 months and one study focused in each lesson on fundamental motor skills [30]. In contrast to our results, children in these two studies improved their motor skills. The first study [30] assessed fundamental motor skills using the Test of Gross Motor Development second edition (TGMD-2) [65] while the second one [34] used the Movement Assessment Battery [6]. Allover, the design of the implementation (study setting vs governmentally-led program, structured vs unstructured physical activity, differences in age) and the methodology used to assess motors skills are quite different between the other trials and our program.

### Predictors of improved outcome

This governmentally-led program was designed to attract as many child care centers as possible and to respect their autonomy, daily functioning and existing time and space limitations. Therefore, the only compulsory components of the program were training of the educators and rearrangement of the built environment, but no precise mandatory demands were made regarding the other components. No information about exact daily physical activity time or the use of any specific curriculum for structured physical activity, the number of trained educators, the provision of a free access to a movement space or a specific type of physical activity equipment, or the organization of a parental information session was obtained. As not all components were implemented by all child care centers, the design allowed us to identify the role of different relevant predictors in one single study.

Thus, different components can be identified as predictors for improved outcomes in previous studies and particularly in the present intervention: 1) The few existing RCT interventions in preschool that had increased effects on motor skill development [29,30,34,35,61], physical activity [2,26-28,33,37], or obesity [26,27,31,33] used a *specific curriculum* to implement structured physical activity (see also above). In accordance with this, as confirmed by a previous study [23], focusing exclusively on free play did not increase objectively measured physical activity. In our program, a specific curriculum might have allowed the achievement of a homogeneous amount of physical activity in the whole intervention group, but this is difficult to implement in daily practice and should not be done at the expense of free playtime [66]. An option might be to formally integrate a specific amount of structured physical activity into the daily routines [37,43,67]. 2) *Educators’ motivation* was judged by the coordinator based on the involvement during and outside of the workshops and meetings and during the on-site visits. As motivation is difficult to measure, its assumed impact on behavioral change or, in our case, on BMI, has been rarely assessed. Similarly, Brown et al. [40] found that child care educator enthusiasm and efforts are directly related to children’s physical activity. In addition, the training of an *additional educator* may have contributed to increase the educator’s motivation in the current study, as the program was lead by two persons. In concordance with previous studies, these findings underline the importance of the educators’ qualification and training [42]. 3) We found that the presence of a *free access to a movement space* and of *mobile physical activity equipment* were related to better motor skills and/or higher physical activity. The presence of a free access movement space favors child-initiated activities and might be in accordance with the findings of Brown et al. [41] who demonstrated that child-initiated activities were associated with higher physical activity levels than educators-initiated ones. Moreover, having free access to a movement space had also an increased effect on motor skills, which confirms the existing relationship between motor skills and physical activity [6,7,25,68-70]. Similarly to our results, the presence of mobile equipment has been shown to increase physical activity in previous studies [16,41]. As suggested by Kreichauf et al. [43], mobile equipment seems to stimulate more physical activity, as it can be used in many different ways, and typically involves games of higher intensity. 4) The importance of *parental involvement* to change children’s lifestyle has been reported in previous successful interventions [44,71-74] but, as far as we are aware, not analyzed as a predictor within a study. In our trial, offering parental sessions was related to an increased effect on motor skills within the intervention group.

### Limitations and strengths

The present study has a number of limitations. To study such a “real-life” program including both precise mandatory components as well as recommendations, evidently leads to a substantial heterogeneity. Moreover, the intervention probably provided an inadequate dose and “type” of physical activity (absence of structured physical activity) to have any beneficial impact on the measured outcomes. To be more relevant in our analyses it would have been interesting to collect precise information about daily physical activity time or structured physical activity implementation. The child care-based investigation of physical activity performed during one single day (mean wearing time of 6 hours) and only during child care attendance, represents another limitation. Considering the literature, a minimum of three days would have been more valuable for this assessment [75]. However, Trost et al. [75] suggest that the variability of this assessment observed over a single day seems to decrease as children get younger. Physical activity measurements restricted to the child care center setting can also offer the advantage to demonstrate potential differences within a defined setting. The motor skill assessment has been previously validated [53]. In our own pilot study (n = 33 children), test-retest correlations were only moderate compared to other motor tests [65,70,76]. The very young age of our sample might explain these relatively low test-retest correlations. The variability at this age in motor performance might be due to the fact that the children are in a period of unsteady motor acquisition. A bigger sample in our pilot study might have strengthened our results. On the other side, this test has the advantage to assess motor skill performance in a relatively short time. Strengths of the study are its design, the investigation of a “real-life” program and its potentially beneficial predictors, the assessment of motor skills and of child care-based objectively measured physical activity and the young age of the children.

### Generalizability

Based on the fact the Youp’là Bouge program was a governmental “real-life” program, was offered to all child care centers without any exclusion criteria and was implemented in both rural and urban regular child care centers, our results should be generalizable. From a practical point of view, the Youp’là Bouge program was feasible and well-received. Child care centers and parents were highly satisfied with the program which allowed its further widespread implementation over the following years outside of a study setting. The assessment also allowed us to identify the predictors that improve the effectiveness of the implementation. Based on the current findings, the program adapted its content and created a label that requires Youp’là Bouge child care centers to comply with the following requirements: 1) 90 minutes/day of physical activity (10 minutes of which structured physical activity); 2) at least one trained educator per child care center; 3) a written physical activity policy to integrate the different intervention components; 4) wherever possible, a free access to an indoor movement space and the purchase of specifically mobile equipment; 5) at least one parental information session/year. The impact of this new and adapted program should be studied in a future trial including new predictors in order to constantly improve existing programs.

## Conclusion

In summary, our results indicate that the “real-life” Youp’là Bouge physical activity program in child care centers did not lead to increased effects in motor skills, BMI, child care-based physical activity or quality of life and confirms the complexity of implementing physical activity intervention outside of a study setting [77]. The novel approach identified potentially relevant predictors within a governmentally-led RCT which helped to adapt the program and the allocation of its resources. The impact of this adapted program should be studied in a future trial including new predictors in order to constantly improve existing programs.

## Abbreviations

BMI: Body mass index; MVPA: Moderate to vigorous physical activity; VPA: Vigorous physical activity; RCT: Randomized control trial.

## Competing interests

Financial disclosures: The authors have no financial relationships relevant to this article to disclose. Conflict of interest: The authors declare that they have no conflicts of interest relevant to this article to disclose.

## Authors’ contribution

AB contributed to the design of the study, acquired, analyzed and interpreted the data, and drafted, wrote, revised the manuscript. He approved the final manuscript as submitted. JB contributed to the conception of the design instruments, assisted in data acquisition, and in drafting the manuscript. He approved the final manuscript as submitted. THK contributed to the conception of the design instruments, and assisted in drafting the manuscript. She approved the final manuscript as submitted. SK assisted in the conception and design of the study, and in drafting the manuscript. She approved the final manuscript as submitted. AL contributed to the conception and design of the study, and assisted in drafting the manuscript. She approved the final manuscript as submitted. CS assisted in data analysis, and critically reviewed the manuscript. He approved the final manuscript as submitted. PMV assisted in data analysis and interpretation, and critically reviewed the manuscript. He approved the final manuscript as submitted. JJP conceived and designed the study, analyzed and interpreted the data, and drafted and revised the manuscript. She approved the final manuscript as submitted. All authors read and approved the final manuscript.
